# Lymphadenoma, or Hodgkin’s Disease

**Published:** 1898-01

**Authors:** W. Horace Hoskins

**Affiliations:** Philadelphia, Pa.


### LYMPHADENOMA, OR HODGKIN'S DISEASE.

By W. Horace Hoskins, D.V.S.,
PHILADELPHIA, PA.

THE subject of this exceptional condition was a collie dog—"Pharaoh"—five and one-half years old, the property of Mr. M. H. F., of Philadelphia, who had raised him from a puppy.

As a puppy he was delicate, and frequently suffered from gastric derangement and irritable skin-eruptions. The care of the animal was excellent, with outdoor daily exercise. The past two years his physical condition was exceptionally good, and his weight ranged around sixty pounds. In fact, he had taken on flesh to an unusual degree and seemed in every way strong and vigorous.

While sojourning in the country during the past summer the only thing noticeable about him was œdematous swellings of the region of the neck; these would disappear in great measure after a few days, and, with his long coat, would hardly be visible. On returning to the city early in October the swellings of the neck became more noticeable and persistent, and when seen, on October 5th, presented enlargements of the thyroids as large as a duck-egg. The thymus, with the accompanying œdema, was as large as one's fist; the pectoral and axillary enlarged, with much œdema along the sternum; the inguinal very much enlarged, with great œdema of the scrotal region and sheath; the temperature elevated; catarrhal discharge from the nose, distressed breathing, and great lassitude; the visible mucous membranes pale and anæmic; appetite good at times, but any exercise seemed to exhaust him. As carcinoma or tuberculosis was suspected, the use of iodide of potash was resorted to, with iron and quinine. On the 13th the distressed breathing became so pronounced that small doses of strychnia were prescribed, which abated the alarming conditions. There being no improvement in the general condition, he was removed to my Sanitarium on the 16th, for the purpose of testing with tuberculin, and the temperature taken for two days prior to injection with the hope of finding a period when it would more nearly approach normal. During this period it rose as high as $104\frac{3}{5}°$ and the lowest point 103°; was injected at 10 P.M. on the 18th, when his temperature was $104\frac{1}{5}°$. During the succeeding twenty-four hours the temperature was taken at ten periods, during which time it ranged from 103° at 8 A.M. on the 19th to $104\frac{2}{5}°$ at 2 P.M. of the same day, receding to $103\frac{1}{6}°$ at 6.30 A.M. on the 20th, thus showing no reaction. A portion of one of the axillary glands was removed for staining and examination for evidence of tuberculois, but the result was negative. His condition grew rapidly worse, and death followed on the 21st.

Autopsy was held a few hours after death. Contents of thoracic cavity normal, save an anæmic appearance of muscular walls of the heart. The liver was very pale in color, spleen enlarged and its pulp liquefied. Intestines very white, anæmic, save the colon and rectum, which were inflamed. Glands of abdominal cavity but slightly enlarged. The lower jugular region, pectoral, axillary, and inguinal regions were the seat of extensive dropsical effusion, while the glandular structures of these regions were hypertrophied to an enormous extent.

Sections of these glands were forwarded to Dr. Robert Formad, of the Veterinary Department of the University of Pennsylvania, who reported the absence of any evidence of tuberculosis, cancer, or sarcoma ; further, that the preparations show only an unusual hypertrophy of the lymphatic structures, but no change or product abnormal to the gland, and constitutes that condition known as lymphadenoma associated with several diseases, often with blood-diseases.

Lymphadenoma, or Hodgkin's disease, has been described under various synonyms, among them pseudo-leukæmia, lymphadenosis, malignant lymphoma, anæmia lymphatica, adenoid disease, adenia, chronic recurrent fever (Ebstein), lineal lymphadenoma, where principal lesion noted is hypertrophy of spleen ; multinuclear leucocytes, lymphatic leukemia of the glands, myelogenic leukemia, when a hyperplastic phenomena of the red marrow of the bones exists. It is described as an enlargement of the lymphatic glands, with progressive anæmia. The pathological lesions, a hyperplasia of lymphoid structures of the body, enlargement of the spleen.

It has been noted in the horse, ox, dog, pig, cat, mouse ; not noted in sheep or goats. Nocard records a case in a cow, where the superficial lymphatic glands were enlarged, and diarrhœa in the later stages.

This case seemed more of the nature of pseudo-leukemia (malignant lympho-sarcoma), of which Frohner records cases in the horse and dog, tending to become chronic, and changing to true leukæmia.

In human pathology the disease is always noted as a chronic condition, usually lasting from two to two-and-one-half years, ascribed to debilitating influences, traumatisms, intermittent fever, syphilis. Symptoms vague : mucous membranes pale ; weakness, lassitude, dyspnœa ; temperature increased. Always chronic ; and sometimes only recognized on autopsy. Prognosis always serious.

Treatment : arsenic, iron, tonics, rich food easily digestible. Termination always fatal.

